# Mixed methods evaluation of the Getting it Right First Time programme - improvements to NHS orthopaedic care in England: study protocol

**DOI:** 10.1186/s12913-017-2012-y

**Published:** 2017-01-23

**Authors:** Helen Barratt, Simon Turner, Andrew Hutchings, Elena Pizzo, Emma Hudson, Tim Briggs, Rob Hurd, Jamie Day, Rachel Yates, Panagiotis Gikas, Stephen Morris, Naomi J Fulop, Rosalind Raine

**Affiliations:** 1 Department of Applied Health Research, University College London, 1-19 Torrington Place, London, WC1E 6BT UK; 2 Department of Health Services Research & Policy, London School of Hygiene & Tropical Medicine, 15-17 Tavistock Place, London, WC1H 9SH UK; 3 Royal National Orthopaedic Hospital NHS Trust, Stanmore, London, HA7 4LP UK

**Keywords:** Orthopaedic surgery, Service reorganisation, Organisational change, Outcomes, Implementation, Health services research, Cost effectiveness

## Abstract

**Background:**

Orthopaedic procedures, such as total hip replacement and total knee replacement, are among the commonest surgical procedures in England. The Getting it Right First Time project (GIRFT) aims to deliver improvements in quality and reductions in the cost of NHS orthopaedic care across the country. We will examine whether the planned changes have delivered improvements in the quality of care and patient outcomes. We will also study the processes involved in developing and implementing changes to care, and professional and organisational factors influencing these processes. In doing so, we will identify lessons to guide future improvement work in other services.

**Methods/design:**

We will evaluate the implementation of the GIRFT programme, and its impact on outcomes and cost, using a mixed methods design. Qualitative methods will be used to understand the programme theory underlying the approach and study the effect of the intervention on practice, using a case study approach. This will include an analysis of the central GIRFT programme and local provider responses. Data will be collected via semi-structured interviews, non-participant observation, and documentary analysis. Quantitative methods will be used to examine ‘what works and at what cost?’ We will also conduct focus groups with patients and members of the public to explore their perceptions of the GIRFT programme. The research will draw on theories of adoption, diffusion, and sustainability of innovation; its characteristics; and contextual factors at provider-level that influence implementation.

**Discussion:**

We will identify generalisable lessons to inform the organisation and delivery of future improvement programmes, to optimise their implementation and impact, both within the UK and internationally. Potential challenges involved in conducting the evaluation include the phased implementation of the intervention in different provider organisations; the inclusion of both retrospective and prospective components; and the effects of ongoing organisational turbulence in the English NHS. However, these issues reflect the realities of service change and its evaluation.

## Background

Orthopaedic procedures, such as total hip replacement (THR) and total knee replacement (TKR), are among the commonest surgical procedures in England [1]. Rates of joint replacement have risen substantially over the past decade [2], whilst orthopaedic referrals from primary to secondary care have increased by 7–8% each year [1]. The first ‘Getting it Right First Time’ (GIRFT) report, published in 2012, recommended changes to NHS orthopaedic practice to improve patient outcomes, and achieve cost savings [1]. NHS England subsequently funded a ‘national professional pilot’ to be implemented by a project team, hosted on behalf of the British Orthopaedic Association (BOA), at the Royal National Orthopaedic Hospital in Stanmore. Senior clinicians visited providers to offer bespoke recommendations about improving care. A ‘dashboard’ of clinical and financial performance data was created for each provider organisation, to inform self-assessment of local services. By July 2015, the GIRFT project team had carried out reviews with 120 NHS Trusts in England. The project highlighted significant variations in practice and outcomes; device and procedure selection; costs; and infection rates [3].

Consequently, the Department of Health (DH) has commissioned a £2.45m, three-year programme to address these challenges initially in orthopaedics, and then across ten other clinical specialities. Building on the visits carried out during the professional pilot, the programme consists of three additional implementation tools, each acting at a different level: regular publication of provider-level performance data via a dashboard (clinical level intervention); tailored written feedback to underperforming providers (top down intervention) and commissioning levers to change behaviour (commissioner level intervention). It seeks to change practice, (e.g. prosthesis choice) in order to improve patient outcomes (e.g. infection rates). Work is underway to enhance patient safety by increasing minimum volumes of complex procedures undertaken by individual surgeons. Regional networks will also be established to increase minimum volumes of complex procedures, and discussions are underway with NHS England and NHS Improvement around improvements to the tariff for purchasing, or commissioning, orthopaedic care. NHS Improvement is currently responsible for overseeing organisations that provide NHS-funded care. Additionally, involvement of relevant professional organisations, including the British Orthopaedic Association (BOA) and the Specialist Orthopaedic Alliance, is being used to encourage change. The programme also has strong links with Lord Carter’s Procurement and Efficiency programme [4].

The GIRFT programme can thus be defined as a ‘complex intervention,’ containing several interacting components, targeting different stakeholders and addressing a number of different behaviours [5]. There is, however, limited evidence to support the proposed approach. Best et al. suggest that the effectiveness of feedback, for example via dashboards, is dependent on the presence of incentives [6], whilst Turner et al. emphasise the importance of a combination of both system (top-down) and distributed (bottom-up) leadership in enabling change [7]. Dixon-Woods et al. also highlight the importance of both ‘hard’ and ‘soft’ incentives for changing behaviour [8]. Whilst there have been reorganisations of a number of acute NHS services in recent years [9, 10], there have been few examples on this scale. Literature about the diffusion of health care innovations highlights the need for more research on the processes by which innovations are initiated, implemented and sustained, and in what contexts [6, 11].

This project aims to contribute to the development of that evidence base by studying in depth the implementation of changes to orthopaedic services. The new DH-commissioned programme, which commenced in January 2016, will continue the work trialled in the national pilot. We will therefore evaluate the intervention as a whole, including the pilot and the work currently being planned. We will focus on outcomes directly relating to patient care, for the commonest elective orthopaedic procedures: THR and TKR [12]. Through this, we will identify lessons to inform future service improvement efforts. In line with Medical Research Council guidance about the evaluation of complex interventions, key goals will be to refine our current understanding of the details and timing of the intervention, as well as the programme theory underpinning its implementation, to inform decisions about which impacts that should be measured [5]. Programme theory sets out the rationale and assumptions about the mechanisms that link an intervention’s processes and inputs to outcomes. Articulation of this theory helps improve the measurement of the intervention’s impacts [13].

### Aim and objectives

The aim of the proposed research is to evaluate the implementation of a complex intervention, ‘the GIRFT programme’, which seeks to improve the quality and cost-effectiveness of NHS orthopaedic care in England. In doing so, we will identify lessons to guide future improvement work in other services.

We will use formative and summative evaluation methods to:

1. Examine the key processes of - and factors influencing - the ongoing development and implementation of planned improvements to orthopaedic services, nationally and locally, including any unintended consequences;
2. Assess whether the GIRFT programme has reduced variations in orthopaedic practice and expenditure, and improved patient outcome measures;
3. Estimate the economic impacts (e.g. cost savings) of these changes; and
4. Explore patient and public perceptions of the planned improvements to care.

## Methods/design

To develop worthwhile lessons for improvement programmes in the future, it is important to establish not just whether process and outcome changes took place, but also how and why they occurred. We will therefore evaluate the implementation of the GIRFT programme, and the impact of specific interventions on outcomes and cost, using a mixed methods design. As part of this, we will explore the interplay between the different implementation tools, acting at different levels, and their impact on both changes in practice and patient outcomes. The following component studies, which are interdependent and will inform one another, are planned in relation to each objective. Qualitative methods will be used to understand the details of the intervention, as well as the programme theory underlying its implementation, and to study the effect of the different ‘implementation tools’ intended to promote changes in practice (Study 1). The research will draw on theories of adoption, diffusion, and sustainability of innovation [11]; its characteristics [14]; and contextual factors at provider-level that influence implementation [15]. Quantitative methods will be used to examine ‘what works and at what cost?’ (Studies 2 and 3). Details of the intervention gathered in Study 1 will be used to inform the choice of variables and outcomes for these studies. We will also use qualitative methods to explore patient perceptions of the planned improvements to orthopaedic care (Study 4). Methods for data collection and analysis are described separately for each study.

To bring together the findings of the four study components, the proposed evaluation will make use of an existing analytic framework that aims to identify key components of major system change, and how they might interact [16]. Drawing on key features of change identified in the implementation literature, the framework distinguishes between the ‘implementation approach;’ ‘implementation outcomes’ (i.e. the adoption of, fidelity to, and sustainability of a given intervention); and ‘intervention outcomes’ (e.g. changes in provision of care or patient outcomes). It will enable us to study the factors that influence implementation of the GIRFT programme (including the nature of the intervention and how its implementation is facilitated), and the potential relationships between these and intervention outcomes, allowing insights into the ‘black box’ of implementation.

### Study 1: Organisational study

To examine the nature and implementation of the changes to orthopaedics, we will conduct a multi-level organisational study of their planning and roll out, using a case study approach [17]. This will include an analysis of the central GIRFT programme and local providers’ responses. Case studies were chosen because this approach allows complex phenomena to be studied in-depth, taking into account both the case (here, organisations providing orthopaedic services) and the context (professional and organisational processes), as well as interactions between the two [17]. The research will focus on the potential implementation tools used to promote change, which are likely to be (1) performance review visits by a senior clinician; (2) performance feedback via ‘dashboards’; (3) letters from the GIRFT project team and other national stakeholders recommending changes to practice, and (4) introduction of ‘best practice’ tariffs for the purchasing of orthopaedic care and related commissioning levers.

Using objective criteria, the evaluation team will identify six case study sites through discussion with the GIRFT project team. Purposive sampling within groups will be used to select: different types of hospital (e.g. district general hospital versus teaching hospital); and different settings (e.g. urban versus rural). Representatives of the wider local health economy will be interviewed where relevant (e.g. clinical commissioning groups (CCGs)). As some for-profit providers with ‘Any Qualified Provider’ (AQP) contracts are not part of the general GIRFT programme, they will also be comparators: representatives of two to three providers will be interviewed to gain their perspective on implementing improvement in orthopaedics. We will identify AQPs providing elective orthopaedic services using internet searches, including the NHS choices website. The selection criteria we apply will ensure the providers sampled reflect different geographies (region of England and urban/rural).

Data will be collected through semi-structured interviews, non-participant observation, and documentary analysis. We will carry out semi-structured interviews and observations at both national (e.g. the GIRFT project team, including understanding and co-developing the ‘programme theory’) and local levels (e.g. providers’ responses to the implementation tools and perceived impact of the approach to implementation, and the tools, on practice). Through the interviews we will examine stakeholders’ perceptions of barriers and facilitators to change. We will also explore at provider level the relationships between the implementation tools, local organisational context, and programme uptake. We anticipate conducting around 70 interviews, 100 h of observations, and analysing relevant documents (e.g. communications with providers). Repeat interviews with providers and the GIRFT project team will be conducted a year apart to assess the impact of the programme over time. A list of interviews is provided in Table 1.

**Table 1:** List of interviews for organisational study (Study 1)

Level	Round 1 interviews	Repeat interviews	Total Interviews
National level			
GIRFT project team	Clinical lead, programme manager, data analyst, Trust lead [*n* = 4]	2	6
GIRFT Provider Enablement Group	Representatives of key stakeholders, e.g. NHS Improvement, British Orthopaedic Association, NHS England, NHS litigation authority [*n* = 5]	2	7
Taskforces	Up to three groups; interviews with chair and key members of group, including commissioners [*n* = 8]	3	11
Provider level			
Each provider site	Up to six interviewees per provider, e.g. service manager, lead consultant, middle manager, board member, e.g. MD or CEO. [*n* = 6x6 sites]	1 x6 sites	7x 6 sites
Comparators outside GIRFT programme	2 providers (AQPs), interview with clinical and/or managerial lead from each provider [*n* = 2]	2	4
Total	55	15	70

Examples of non-participant observations at the national level will include attending the GIRFT project team’s planning meetings, as well as dissemination and educational events. Observations at the provider level will include attending one-to-one feedback sessions provided by the GIRFT project team and locally initiated responses to change, e.g. performance improvement forums organised around the ‘dashboard’ and other implementation tools.

We will also collect documentation about the GIRFT programme at both national and local level in order to capture how the programme is planned, communicated to providers, and local responses from providers. These documents will cover how the programme was developed and the processes of implementation (e.g. GIRFT programme documentation; meeting minutes; letters and other communications with providers; performance reports; dashboards; and local providers’ documented responses to the programme). Project documentation will be collected over the lifespan of the evaluation. It will inform the planning and conduct of the interviews and non-participant observations in Study 1, as well as the proposed quantitative analysis (Study 2).

Interviews will be digitally recorded for professional transcription in full. Field journals for recording notes will be kept by the researchers. Data will be analysed thematically, combining inductive (ideas emerging from the empirical data) and deductive approaches (with reference to the innovations and implementation literature) [18]. Initial analysis and category building will be conducted by the researchers and will include category mapping and constant comparison. Cross case comparison will be used to compare and contrast findings at different levels within the same case study (e.g. differences in the response of senior executives and service level within providers) and the same level across different case studies (e.g. variation in responses of providers, role of CCGs). Validity will be assessed in relation to Patton’s four criteria of validity in qualitative research: verification, rival explanations, negative cases and triangulation [19]. Analysis of documentary evidence will focus on documents covering planning, events and progress of the GIRFT programme, to build an understanding of the development and implementation of the programme and providers’ responses, as well as identifying events that may have influenced progress of the work.

### Study 2: Quantitative study

The quantitative analysis will examine the relationship between the introduction and delivery of different components of the ‘intervention’ (i.e. the implementation tools) and intended changes to care processes and patient outcomes. The methods will be developed in detail during the project and will draw on information about the intervention gathered during the organisational study (Study 1).

The quantitative analysis will be based on longitudinal organisational data with NHS provider or hospital as the primary unit of analysis. It will comprise (1) data from the GIRFT project team that describe the implementation tools and (2) process and outcome measures from linked anonymised patient-level data from routine sources. The analysis will seek to relate variation in the implementation tools (independent variables) with changes in measures of process and outcome (dependent variables). The final choice of variables will be based on findings from the multi-level organisational study (Study 1).

The three indicative implementation tools are likely to comprise performance review visits by a senior clinician; dashboard distribution; provider letters (both trust-level); and tariff pricing levers (national). Details of the interventions and their timing will be confirmed during the Study 1 interviews with the GIRFT project team. However, implementation is likely to occur during the period 2015–2017 and, in the case of dashboards and letters, vary in timing between trusts.

Measures of process and outcome - the dependent variables - are likely to be derived from routine linked patient-level data (Hospital Episode Statistics (HES) admitted patient care; National Joint Registry (NJR); Public Health England surveillance data; and national patient-reported outcomes (PROMs) programme for elective surgery) and may comprise re-admission rates; rates of revision surgery; surgical site infection; length of stay; patient reported outcomes; type and cost of prosthesis; and surgeon/consultant volume. Information about the use and cost of loan kits by providers will be provided by GIRFT. In addition, linked patient-level data will also provide information on potential confounders such as socio-demographic characteristics (age, sex, deprivation and ethnicity) and comorbidity. Data will be linked at the patient level by an encrypted identifier (encrypted HESID); no patient identifiable data will be required. Information about surgeon volumes is included in NJR data. It is anticipated that full data will cover the period from April 2009, when England's national PROMs programme started, to March 2018. Patient-level data and trust-level data on interventions will be linked by the HES trust provider code.

The approach to the analysis will also be dependent on the findings of the multi-level organisational study (Study 1). Descriptive analysis will map the timing and duration of the implementation tools in trusts with the periods of availability of linked patient-level data to identify differences in timing within trusts and the number of monthly periods of pre- and post-implementation patient-level data that will be available for analysis. It is likely that the chosen analytical approach will involve multivariable regression that tests for changes in trends for the different process and outcome measures in response to the interventions. Analyses will need to allow for non-independence (e.g. at trust and/or surgeon level); adjustment for potential confounding; specification of modelled effect for intervention (e.g. change in trend/step-change, wash-out period/delayed response); and control. Control is likely to combine within-trust control (before:after intervention) and potentially between-trust control (differences in timing in intervention sites and inclusion of non-intervention sites, e.g. independent providers of NHS orthopaedic surgery). Tariff pricing levers, as a national intervention, will not allow between-trust control. Interpretation of results will be sensitive to the potential for residual confounding and other methodological limitations, e.g. ‘contamination’ in control sites.

A statistical analysis plan will be produced as part of Study 2. The plan will determine the analytical approach in greater detail and will draw on both the qualitative study and initial mapping and analyses from the quantitative study (variation, frequency and completeness of proposed independent and dependent variables). In particular, the plan will consider the extent that it is possible to differentiate between the impacts of the different implementation tools given their likely overlap in timing and ensure that sufficient periods of post-introduction data are available to allow an assessment of any impact. Initial analyses will examine data on the implementation tools and on the process and outcome measures separately. Data will only be combined once the analysis plan has been approved by the study steering group. The timing of the interventions and the availability of data within the time-frame of the project will also determine which analyses will be undertaken during the project. In the event that the required data for this component of the evaluation are not available during the lifetime of this grant, we will apply for additional grant funding to continue the work.

### Study 3: Economic study

To assess value for money, we will identify the difference in costs and the difference in benefits of implementing the GIRFT programme compared to the situation ‘in absence of intervention,’ using a similar methodological approach to that described above for Study 2.

Three main cost categories will be explored: (1) the extra cost of GIRFT implementation tools (e.g. the cost of performance review visits by a senior clinician); (2) the extra cost incurred by providers to implement recommendations and sustain change (e.g. ring fenced units); and (3) reduction in costs related to patient outcomes (e.g. reduced expenditure on loan kits). Benefits of the GIRFT programme could include health outcomes for patients (e.g. reduced surgical site infections). The GIRFT project team’s partnership with the NJR will allow us to explore the relationship between implant cost and effectiveness, measured by implant longevity and revision rate.

Data on the following costs will be examined: (1) the extra cost of GIRFT tools will be assessed using the data, documents and invoices provided by the GIRFT project team and where not available costed using marked prices; (2) the extra cost incurred by providers to implement recommendations will be gathered directly from Trusts or using data from the literature; (3) reduction in costs related to outcomes will be assessed using NHS costs, payment by results costs for length of stay, other costs (for staff, consultations etc.) and data from literature where the above are not available.

### Study 4: Patient and public focus groups

The planned improvements to orthopaedic care across the country have been clinically led. To inform the development of future improvement programmes, we will explore patient and public perceptions of the proposed changes, and the desired outcomes. To do this, we will conduct three focus groups, each with 6–8 participants. Two of the three groups (Groups 1 and 2) will involve patients who have undergone an elective hip or knee replacement in the two years prior to the start of this study (Jan 2016–Jan 2018). Group 3 will include patient representatives who have not undergone joint replacement surgery, but who are in the age bracket most likely to receive this type of intervention (age 60+) [20]. As participants may not be aware of the proposed changes to orthopaedics, the researchers will provide a brief lay language overview of the GIRFT programme at the start of each focus group.

Participants for Groups 1 and 2 will be recruited via patient representative groups. This will include the British Orthopaedic Association Patient Liaison Group and groups within NIHR CLAHRC North Thames provider organisations in North Central and East London, Bedfordshire, Essex and Hertfordshire. Group 3 will be recruited via hospital based patient involvement groups, and local organisations such as Health Watch in the NIHR CLAHRC North Thames area.

During the focus groups, we will explore participants’ views about (1) the intended changes to practice and (2) the intended improvements to patient outcomes. At the start of the focus groups, the researchers will give participants a brief overview of the planned changes to orthopaedics. Focus groups will be digitally recorded for professional transcription in full. Fieldwork notes will also be kept by the researcher. As in Study 1, data will be analysed thematically, combining inductive and deductive approaches [18].

### Synthesis of quantitative and qualitative approaches

The qualitative and quantitative components of the evaluation will be brought together in three ways. First, a key goal of the organisational study (Study 1) is to understand the programme theory underlying the GIRFT approach. The findings will therefore directly inform both the analytic design and final selection of variables for the quantitative and economic studies (Studies 2 and 3). Details of the intervention components and their timing will also be confirmed during the Study 1 interviews with the GIRFT project team.

Second, the dissemination of our findings will include qualitative data about the implementation of the GIRFT programme and patient/public perceptions of the intervention, as well as quantitative data describing the impact of specific interventions on outcomes and cost.

Finally, we will bring together the findings of all the study components, using Fulop et al’s framework for analyzing major system change [16]. This will enable us to identify the key components of the GIRFT programme, and the factors influencing its implementation, as well as how these may have interacted.

### Patient and public involvement

We have worked with the NIHR CLAHRC North Thames Patient and Public Involvement/Engagement Officer to seek the views of patients and the public on the proposed evaluation, via the CLAHRC’s lay Research Advisory Panel and the British Orthopaedic Association Patient Liaison Group. Their feedback has been used to refine the protocol and ensure appropriate consideration of the patient perspective. Through this, we have identified three orthopaedic patients who have agreed to join our Patient and Public Reference Group, together providing a range of perspectives on care. This group will have a collaborative role in refining research design, interpreting findings and disseminating results and will meet physically three times, as well as being involved in the interim via email/telephone/webinar. This will include ensuring that patient priorities are adequately represented in Study 4, the patient focus groups. We will provide training to members where necessary, as well as appropriate reward and reimbursement for their contribution and expenses incurred in taking part.

## Discussion

In this protocol, we describe a proposed evaluation of planned changes to NHS elective orthopaedic care in England. The Getting it Right First Time project (GIRFT) aims to deliver improvements in quality and reductions in the cost of orthopaedic care across the country. The approach will in due course be rolled out across ten other NHS surgical specialities. We will conduct a mixed methods evaluation of the changes in orthopaedics, using formative and summative approaches. Methods will include documentary analysis; one to one interviews with a range of stakeholders; analysis of performance and cost data; and focus groups with patients and members of the public.

The organisational component (Study 1) will permit the identification of factors that are influential in the process of change, such as key obstacles to and enablers of change at the professional and organisational level, and how best to engage with these. The provider-level case studies will also permit identification of both generalisable and context-specific lessons. The quantitative and economic studies (Studies 2 and 3) will describe changes in the provision and cost of orthopaedic services, and what impact these changes have on both the organisation of care and patient outcomes. The patient focus groups (Study 4) will enable us to describe the views of patients and the public about service change of this kind. Combining the qualitative and quantitative research elements, for example by using the findings of the organisational study (Study 1) to inform the design of the quantitative study (Study 2), will permit an examination of how change activities, contextual factors, and outcomes interrelate.

The evaluation faces a number of wider methodological and practical challenges.

First, the intervention was rolled out at slightly different times in different provider organisations, as visits by the GIRFT project team in the national professional pilot took place in a phased way between September 2013 and July 2015 [3]. Therefore, as each provider organisation has different ‘before’ and ‘after’ phases, it is possible that external factors might have influenced each organisation differently. Also, conclusions drawn about the relationship between the intervention and any improvements seen are less clear in ‘before’ and ‘after’ studies. It is possible that any improvements that occur could have happened anyway and not as a direct result of the policies implemented. Without randomisation, it is also harder to rule out potential biases and gaming. However, these complexities reflect the ‘real world’ nature of service change, and are thus commonly faced in evaluations of this kind [21].

Second, the evaluation also includes both retrospective and prospective components. This has the potential to impact the organisational component of the research (Study 2). For example, the planning and implementation of the national professional pilot was not observed and interviewees’ recollection of events will naturally vary. Ensuring suitable representation of stakeholders—based on documentary analysis—and applying Patton’s validity criteria [19] in the analysis will be key to ensuring that important lessons are not missed.

Finally, the GIRFT programme has been implemented during a period of significant turbulence in the NHS, encompassing both the changes associated with the Health and Social Care Act in 2013 and now a period of unprecedented financial challenge. However, our proposed methods will allow us to capture some of the influences of such turbulence, and how these might be dealt with when sustaining change. The distraction and uncertainty brought by changes in associated organisations, such as commissioning structures, is common in many settings, meaning the research may have relevance in a wide range of settings.

In conclusion, this evaluation will provide an opportunity to understand better what works, at what cost, as well as how changes of this kind are implemented and sustained. Reviews indicate that more research is needed to understand drivers, processes, and outcomes when implementing such large-scale changes [6, 11]. We will identify lessons to inform the organisation and delivery of future improvement programmes, both within the UK and internationally, to optimise their implementation and impact.

## Abbreviations

AQP: Any Qualified Provider
BOA: British Orthopaedic Association
CCG: Clinical commissioning group
DH: Department of Health
GIRFT: Getting it Right First Time
HES: Hospital Episode Statistics
NHS: National Health Service
NJR: National Joint Registry
PROMs: Patient-reported outcome measures
THR: Total hip replacement
TKR: Total knee replacement
